# Investigation of Damping Properties of Natural Fiber-Reinforced Composites at Various Impact Energy Levels

**DOI:** 10.3390/polym16243553

**Published:** 2024-12-20

**Authors:** Ercan Şimşir, Yelda Akçin Ergün, İbrahim Yavuz

**Affiliations:** 1 Department of Automotive Engineering, Faculty of Technology, Afyon Kocatepe University, Afyonkarahisar 03200, Turkey; iyavuz@aku.edu.tr; 2 Department of Metallurgy and Materials Engineering, Faculty of Technology, Afyon Kocatepe University, Afyonkarahisar 03200, Turkey; yeldaakcin@aku.edu.tr

**Keywords:** natural fiber, low-speed impact test, laminated composite, XPS, EPS

## Abstract

Natural fiber-reinforced composites are composite materials composed of natural fibers, such as plant fibers and synthetic biopolymers. These environmentally friendly composites are biodegradable, renewable, cheap, lightweight, and low-density, attracting attention as eco-friendly alternatives to synthetic fiber-reinforced composites. In this study, natural fiber-reinforced polymer foam core layered composites were produced for the automotive industry. Fabrics woven from goat wool were used as the natural fiber. Polymer foam with expanded polystyrene (EPS) and extruded polystyrene (XPS) structures was used as the core material. During production, fibers were bonded to the upper and lower layers of the core structures using resin. The hand lay-up method was used in production. After resin application, the samples were cured under a heated press for 2 h. After the production was completed, the material was cut according to the standards (10-20-30 Joule), and impact and bending tests were conducted at three different energy levels. The experiments revealed that at 10 J, the material exhibited rebound; at 20 J, it showed resistance to stabbing; and at 30 J, it experienced penetration. While EPS foam demonstrated higher impact resistance in the 10 J test, it was found that XPS foam exhibited better impact resistance and absorption capabilities in the 20 J and 30 J tests. Due to the open and semi-closed cell structure of EPS foams and the closed cell structure of XPS foams, it has been concluded that XPS foams exhibit higher impact resistance and better energy absorption properties

## 1. Introduction

With all the technological developments achieved in the 21st century, humanity has become increasingly sensitive to the environmental damage caused by these advancements. Particularly, the rapid increase in waste production poses threats to human health, the environment, and our planet. The situation has been exacerbated by the recent population growth, with global waste production expected to reach 3.4 billion tons by 2050 [1]. The release of unwanted products considered as waste is an unavoidable situation in many processes. It is known that approximately 7–9 billion tons of waste are produced annually worldwide [2]. At this point, the scientific world has focused on research into sustainable solutions, with materials that are biodegradable yet meet stringent service demands gaining importance. Natural fiber-reinforced composites stand out as an important area of research in materials science [3,4,5,6]. Approximately 2.4 million tons of sheep and goat wool are produced annually, with only a quarter of this amount being used in textile production [7,8,9]. The remaining wool is considered waste material that needs to be disposed of somehow. This disposal process typically involves incineration or destruction options, which can lead to significant environmental problems. Waiting for it to biodegrade is time consuming [10]. The most practical solution for waste management is to reuse waste materials and reintroduce them into production instead of using natural resources. Such a recycling process offers various advantages, such as reducing pollution, eliminating or reducing waste sent to landfills, and preserving raw materials [11,12,13,14,15,16]. Therefore, the recycling of manufactured products is an important issue both economically and environmentally.

Composite materials are novel materials obtained by combining at least two components, reinforcement and matrix, at a macro level. The properties of the reinforcement are enhanced by the matrix that binds these reinforcements together, thus facilitating the entire material design process. In recent years, they have been used in a variety of industries, including automotive, energy, transportation, and aviation, because of their high specific strength values. In these fields, where polymer matrices and reinforcements like carbon, glass, and aramid fibers are frequently used, the usage of natural fibers has grown in popularity. Relatively high specific strength, affordability, formability, low weight, sustainability and environmental friendliness, biocompatibility, and robust fatigue and corrosion resistance are some of the benefits of natural fibers over conventional substitutes [15,17,18]. Fiber generated from vegetable or animal sources is referred to as natural fiber. One bioproduct that develops on the bodies of animals like sheep and goats is wool, which is produced continuously over the course of the animal’s life. The thickness, length, and curl of the fibers determine how these wools are used. Because of its thermal properties, wool is frequently employed as an insulating material [19]. In addition to its thermal efficiency, wool has excellent hydrophobic and hydrophilic qualities and is remarkably durable. Wool fibers are ideal for polymer composites that need to be extremely strong since they are extremely fatigue resistant and can stretch up to 20,000 times before breaking. Wool can be used with asbestos in fire-resistant composites since it is also fire resistant. Wool has been thoroughly studied for its usage in military protective gear due to its strength, flexibility, and longevity [20,21,22,23,24]. 

A review of the literature reveals that natural fiber-reinforced polymers, or NFRPs, are becoming more and more popular in both the academic and industrial sectors. Their natural fibers’ qualities have led to their employment in a wide range of applications, including automotive (e.g., door panels, console systems, and bumper reinforcements) [25,26,27] aerospace (e.g., interior panels, lightweight structures, and reinforced composite components), [28,29,30] furniture, musical instrument making (e.g., guitar bodies, violin backs) [31,32] and it has also found use in sports equipment (e.g., tennis rackets, ski boards) [33]. 

The automotive sector uses NFRP composites for a number of purposes, such as seat backs [34,35,36], floor and door panels [37,38,39], car floor coverings and ceilings [40,41,42], brake pads [43,44,45], and luggage compartments [46,47]. They are employed in both interior and exterior components. The protection of passengers is an extremely important issue, especially in transportation, leading to the constant development of new shock absorbers for transportation applications. Sandwich structures are leaders in this field due to their high bending strength-to-weight ratio and energy absorption capacity. They consist of two thin outer layers covering a thick, lightweight core. This structure is capable of providing very high flexural strength and torsional stiffness [48] while reducing weight compared to solid monomaterial based designs. 

Upon reviewing the literature, it becomes evident that there are numerous studies examining the energy absorption capabilities of sandwich structures. Some of these studies have focused on the contribution of the change in the microstructure of the core material, or in the case of [49,50], the change in the shape of the core material to the mechanical properties of the sandwich material and therefore to its energy absorption capacity. Additive manufacturing is highlighted in studies where changes in the shape of the core material are considered [51,52,53,54]. Two separate sandwich composites consisting of polypropylene and carbon fiber layers on a polypropylene core were produced, and which of these materials would have the better energy absorption capacity was investigated both experimentally and with the finite element method [55].

Given the significance of passenger safety in transportation, this study aims to investigate the energy absorption properties of a sandwich composite material obtained using natural goat wool and two different polymer foam cores. In the study, a wide literature review was conducted and very few studies were encountered in the literature with goat wool. This study aimed to evaluate the impact and damping behaviors of goat wool reinforced composites at different energy levels. Another aim of the study was to compare the behaviors of core materials with different properties and densities against impact energy. For this reason, two different polystyrene-based foam materials, XPS (extruded polystyrene) and EPS (expanded polystyrene) foams, were used in the study. Therefore, the study will produce a material that is both cost-effective and environmentally friendly and will contribute to the literature.

## 2. Materials and Methods

**Figure 1:**
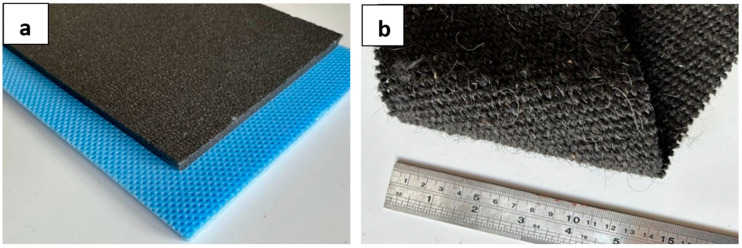
(**a**) EPS (black color) and XPS (blue color) core material. (**b**) Goat wool fabric.

Sandwich structures are composite materials obtained with a core and layers placed on this core. In this study, two different foam materials were used as core material: XPS (extruded polystyrene) and EPS (expanded polystyrene) (Figure 1a). Fabrics woven from goat wool were used as the layer (Figure 1b). Goat wool is a warm, soft and durable type of fiber and can be used in many clothing and home textile products. Goat wool may have different properties than sheep wool. For example, goat wool may contain finer fibers, which can create a softer and lighter fabric [56].

Porous structures have many areas of use in many sectors due to their lightness and excellent energy absorption capabilities. Factors such as the air gaps contained in porous structures, the shape, size, and distribution of the gaps all directly affect the energy absorption capabilities of these structures [57]. EPS (expanded polystyrene) foam is produced by expanding polystyrene granules and then shaping them in a mold. It is a lightweight polymer that is usually produced for various areas of use such as foam cups, packaging materials, and structural insulation. XPS (extruded polystyrene) foam is a polymer foam material produced by extrusion under high temperature and pressure. During this process, polystyrene granules are melted and passed through a series of molds on the extrusion line. These molds allow the material to take the desired shape, and the formation of the cell structure is ensured by adding foaming agents. As a result, a dense, lightweight, waterproof, and insulating material is obtained [58]. One of the main benefits of EPS foam is its affordability. It is also resistant to moisture absorption. However, it has a lower compressive strength compared to XPS.

**Figure 2:**
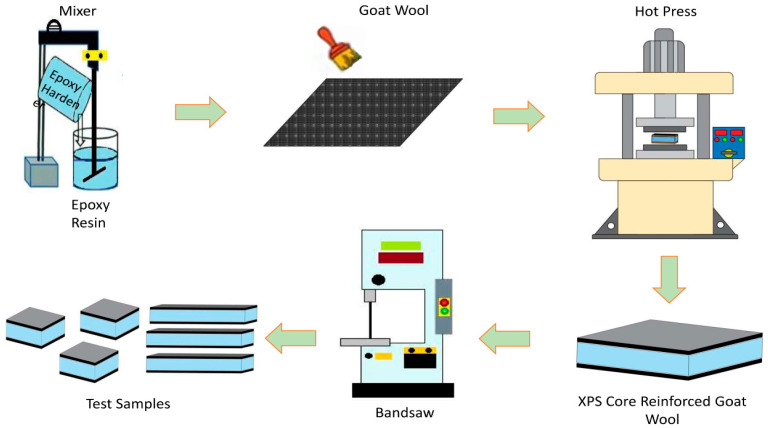
Schematic representation of the sample production process.

Figure 2 schematically illustrates the production process of the composite samples. After cutting the fabric and cores to the desired dimensions, the wool fabric was glued to both surfaces of the core materials as a layer to prepare the samples using the hand lay-up method. MGS LR 285 epoxy resin and LH 285 epoxy hardener were used for sample production. Then, the samples were subjected to a curing process in a hot press under 2 bar pressure. The curing process was completed in two hours at 40 °C. After the curing process was completed, the produced test samples were cut to the dimensions in accordance with the standards. Low-speed impact test samples were prepared in accordance with the ASTM D3763 standard and cut to the dimensions of 100 mm in length, 100 mm in width, and 22 mm in thickness. Three-point bend test samples were prepared in accordance with the ASTM D7264 standard and cut to the dimensions of 125 mm in length, 13 mm in width, and 22 mm in thickness using a band saw.

### Impact Test and Three-Point Bending Test

**Figure 3:**
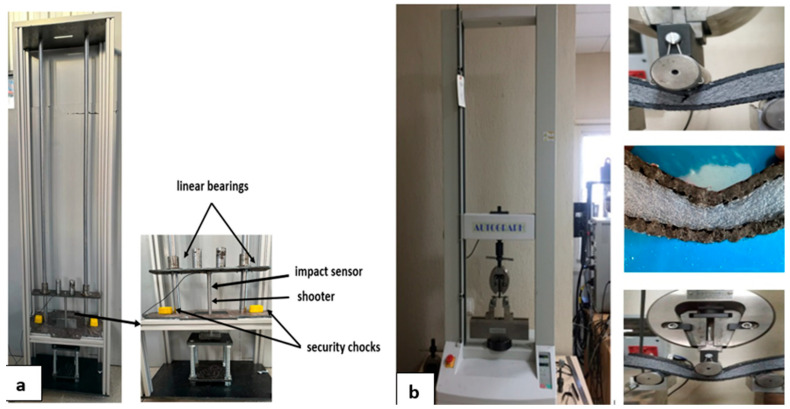
(**a**) Low-speed impact test device. (**b**) Three-point bending test device.

The impact test device used in the study, which operates according to the free-fall weight principle, is shown in Figure 3a. The striking tip of the test device is in the shape of a steel hemisphere and has a diameter of 16 mm. The weight of the striking tip used in the tests is 6.3 kg. Tests were conducted at room temperature. The maximum drop height of the device is 1950 mm. The experiments were carried out with three different impact energies: 10 J, 20 J, and 30 J. The tests were performed according to the ASTM D3763 standard [59,60].

The three-point bending test was conducted using a Shimadzu Autograph tensile device with a capacity of 10 kN. The feed rate was set at 1 mm/min. Three-point bending tests were carried out in accordance with the ASTM D7264 standard [61,62]. Figure 3b shows the test device used in the test and the images of the samples during the test.

## 3. Results and Discussion

### 3.1. Impact Experiment

**Figure 4:**
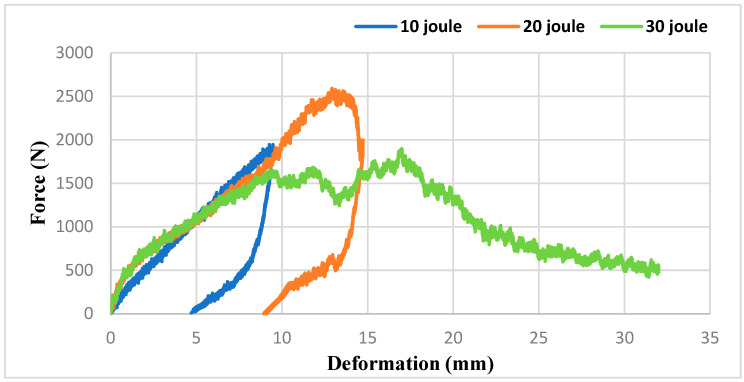
Impact test results of EPS sandwich composites.

**Figure 5:**
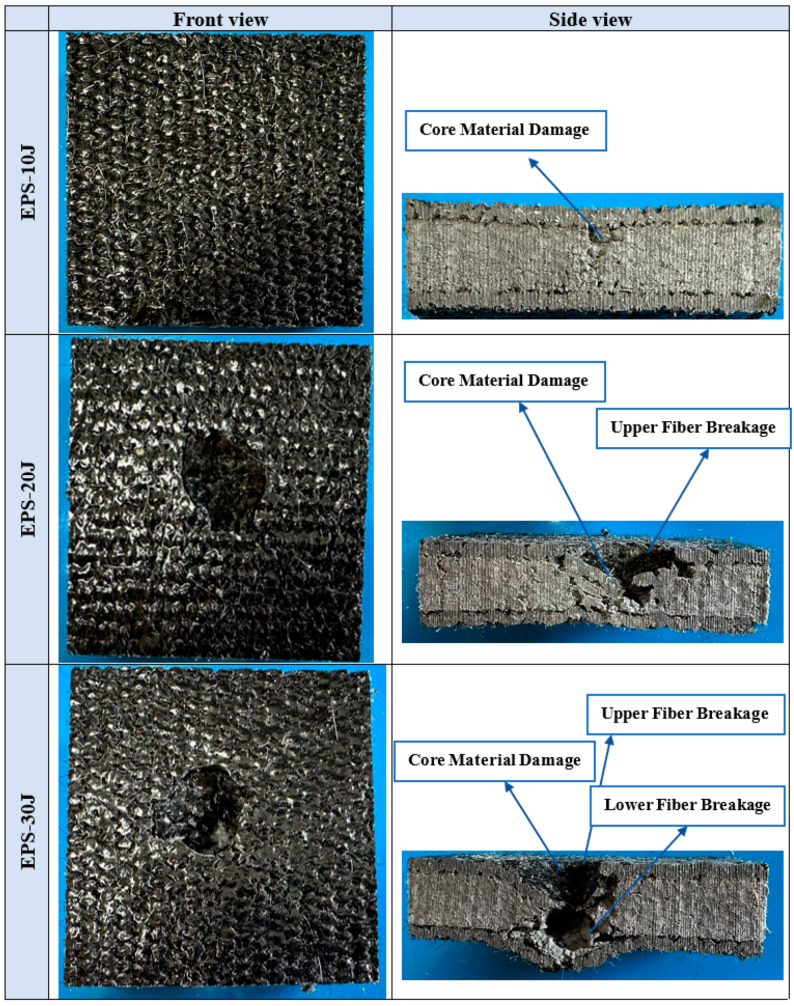
Macro images of EPS sandwich composites after impact test.

Figure 4 illustrates the impact test results of sandwich composites produced with an EPS core under 10, 20, and 30 joules of energy. In force–deformation graphs, deformation is expected to increase with increasing impact energy. Upon examining the impact test results of samples produced with an EPS core, it is observed that the material withstands the 10 J impact without penetration. Macro images taken from the sample after the impact (Figure 5) reveal no damage on the upper and lower surfaces from the 10 J impact. However, a small damage is observed on the core near the upper layer upon cross-section inspection. With 20 J and 30 J impacts, the upper surfaces of the samples are completely pierced, and the cores are damaged. Additionally, with the 30 J impact, damage extends to the back surface of the sample, resulting in penetration, whereas the 20 J impact causes core damage without affecting the lower outer layer.

**Figure 6:**
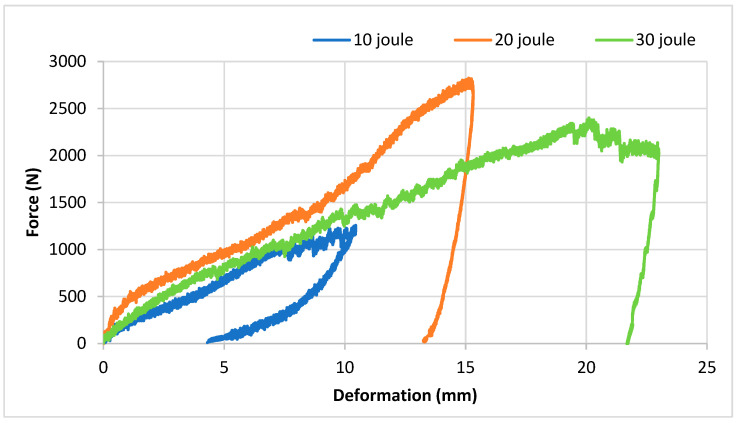
Impact test results of XPS sandwich composites.

**Figure 7:**
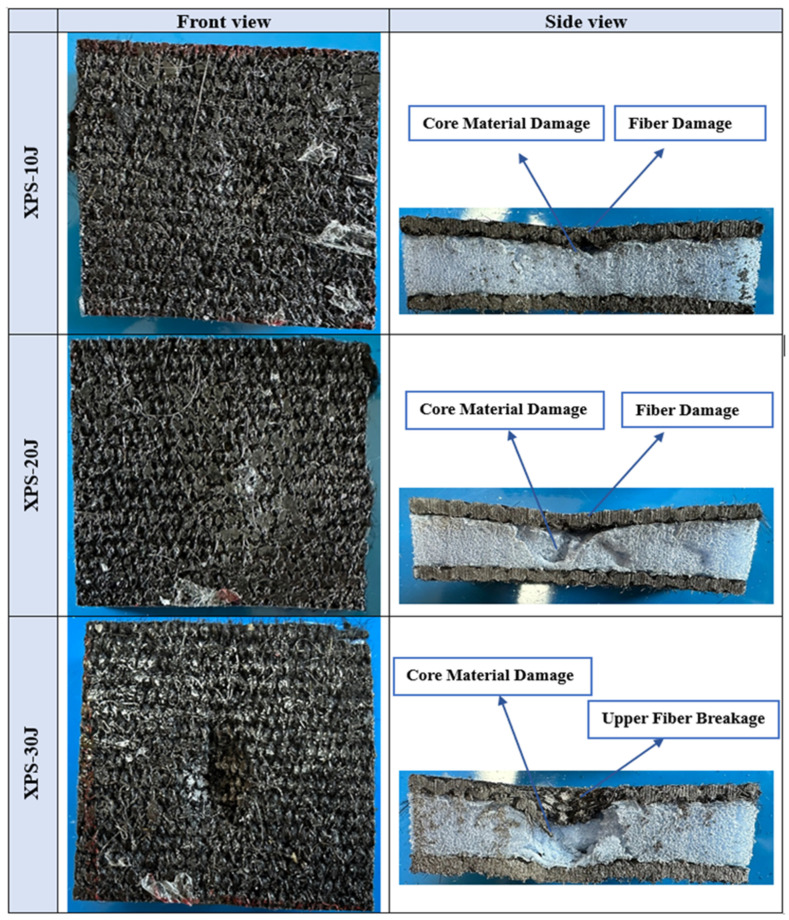
Macro images of XPS sandwich composites after impact tests.

Figure 6 depicts the impact test results of our sandwich composites produced with an XPS core under 10, 20, and 30 joules of energy. At the 10 J energy level, a relatively low force was applied to the sample and the maximum force was around 1000 N. No deformation occurred and resulted in the striking tip rebounding. At the 20 J energy level, the maximum force was around 2700 N. At this level, a sudden drop was observed after the material reached its maximum strength. This drop indicates that damage began on the sample. As a result of the detailed examination of the macro images in Figure 7, it was determined that the beginning of damage was observed in the fibers in the front layer and that the core material was significantly damaged. At the 30 J energy level, the maximum force was around 2500 N and was slightly lower than the 20 J energy. This is thought to be due to the perforation of the front layer. When the cross-sectional view in Figure 7 was examined, it was seen that a larger plastic deformation was caused on the core material. 

Figure 7 shows macro images of damages occurring after impact tests at 10 J, 20 J, and 30 J energy levels of XPS core composites. When the front views are examined, it is seen that the extent of damage in both the core material and fiber layers increases with the increase in impact energy. While very little deformation is observed in the core material at the 10 J energy level, no damage is observed in the fiber layers. At the 20 J energy level, a more pronounced deformation is observed in the core material and damage occurs in the form of a slight collapse in the front layer. No fiber breakage is observed. At the 30 J energy level, fiber breakage occurred in the front layer and serious crushing and fragmentation damage occurred in the core material. No fiber breakage or deformation was observed in the lower layer. When the side views are examined, it is seen that the extent of damage in both the core material and fiber layers increases with the increase in impact energy.

Based on the impact test results conducted with 20 J and 30 J energy levels, it is evident that XPS structures exhibit higher impact resistance. When a material demonstrates very high impact strength and minimal deformation, it indicates a low ability to absorb impact. For effective damping, the applied impact force should not decrease immediately but should instead proceed horizontally, resulting in increased deformation [52]. This approach is important for impact absorbing materials because sudden deceleration can cause high stresses on the material and cause different damages on the material. Constant force and large deformation behavior show that an object can absorb more kinetic energy. For example, steel materials and designs are used with large displacement capacity and elasticity properties for vehicles to absorb kinetic energy during an accident. Thus, the speed of people or objects is gradually slowed down and energy absorption is optimized.

**Figure 8:**
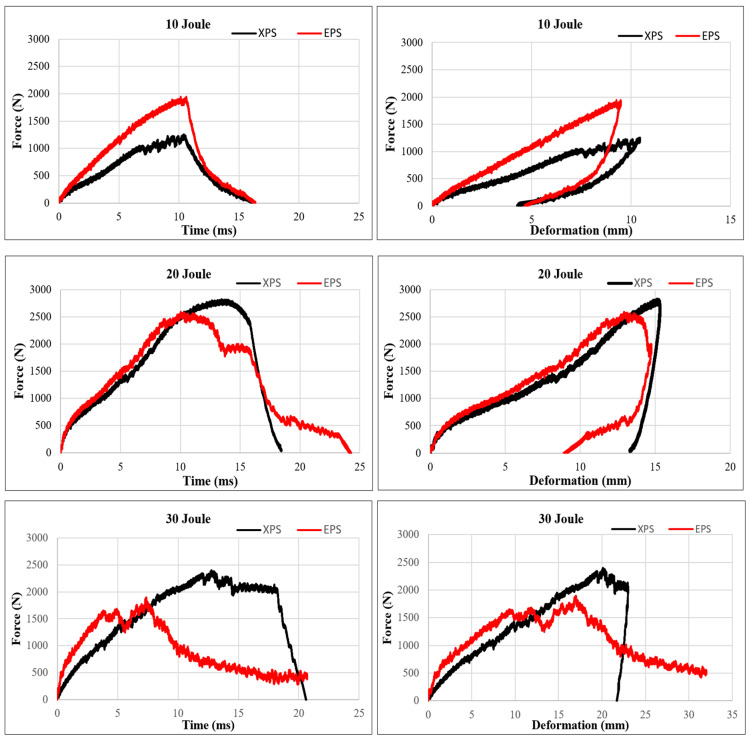
Graphs of impact strength of EPS and XPS cores depending on deformation and time at different energy levels.

When the force–time curves of the XPS structures in the experiments conducted with 20 J and 30 J are examined in Figure 8, it is evident that the curve remains horizontal after the applied force reaches its maximum. Particularly in the experiment conducted with 30 J, a brittle fracture (perforation) was observed in the EPS core structure, while a similar brittle fracture (perforation) occurred in the XPS core structure between 13 and 19 times. The force does not immediately decrease between seconds and remains constant horizontally. During this period, the sandwich structure continues to support loads and dissipates impact energy through damage to the upper layer. In terms of impact resistance behavior, this is highly desirable as it leads to a softening of reaction forces (and therefore accelerations). This observation is further confirmed by the force–deformation graph. Notably, when it reached its peak, there was approximately 19 mm of deformation in the XPS foam, while the EPS core structure experienced a deformation of 16 mm.

### 3.2. Bending Test

**Figure 9:**
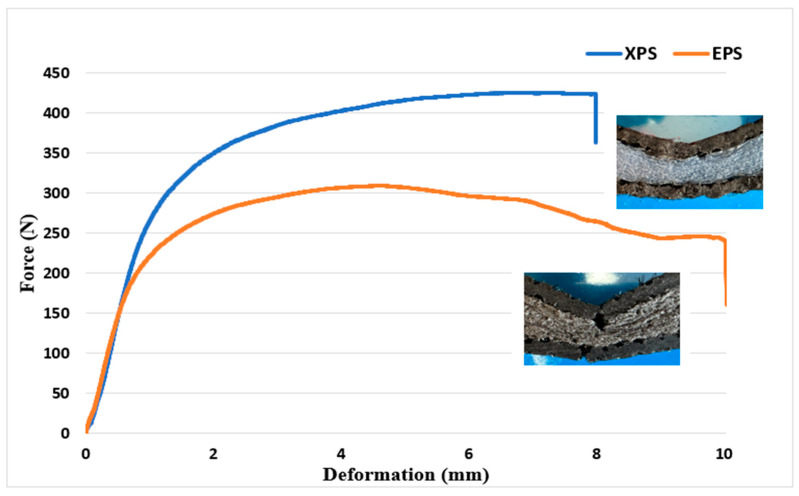
Three-point bending test results.

The results of the three-point bending test are presented in Figure 9. Upon reviewing these results, it becomes evident that the bending strength of the composite produced with an XPS core is higher. In sandwich structures, the interface is a critical component that greatly affects the mechanical properties of the sandwich composite, because the interface provides the connection between the outer surface material and the core material. This connection is critical for structural integrity. A strong interface connection ensures that the loads are distributed homogeneously to the structure and increases the material strength. In addition, the loads in the sandwich composite structure are generally transferred through the outer surface material. The interface ensures that the loads are effectively transmitted to the core material. This is important for optimizing structural performance and increasing the load carrying capacity. When the bending test results, impact test results, and damage images are examined, it is seen that the sandwich composite material obtained with an XPS core and goat wool fabric generally exhibits better mechanical behavior. In the damage mechanisms, no layer separation was observed for both cores. This shows that the reinforcement and the cores are compatible. While the composite produced with the EPS core showed better impact resistance in the test conducted with 10 J energy, the XPS core behaved more durably in the tests of 20 J and 30 J. The mechanical properties of polymer materials can change with the deformation rate. Therefore, the EPS foam behaved differently at low and higher deformation rates. However, the higher compressive strength and density of the XPS foam resulted in better results in high-energy experiments. In addition, the closed pore structure of the XPS foam increased the energy absorption ability of the sandwich structure.

Closed porous structures undergo elastic deformation under the force applied to them. During this deformation, the gas inside the pores compresses and stores energy. Elastic deformation allows the porous material to return to its original form and provides minimal permanent deformation during the energy absorption process. Another important feature of closed pores is that they can distribute the force applied to them throughout their volume due to the interconnection of the cell walls. The regular and closed structure of the pores prevents the concentration of loads at certain points, thus distributing and absorbing the energy homogeneously throughout the material. This situation can be explained by the horizontal course of the force–deformation curve in the three-point bending test (Figure 9) [63,64].

## 4. Conclusions

In this study, two different sandwich composites were prepared using goat wool fiber fabric and EPS and XPS foam cores. The impact absorption capabilities and bending strength of these structures were investigated under 10 J, 20 J, and 30 J energy levels. According to the results obtained,

- No damage was observed on the upper and lower surfaces of the EPS core composite material sample tested at 10 J. Only a small amount of damage was observed in the part of the core close to the upper layer. The upper surfaces of the samples were completely pierced, and the cores were damaged with the 20 J and 30 J impacts. The sample was pierced at a 30 J impact.
- As a result of the impact tests of the sandwich composites produced with the EPS core, in the 20 J experiment, damage was observed on the core and the upper surface where the impact was applied, but no damage occurred on the lower surface. In the impact test performed at 30 J, the upper layer was pierced and the core was damaged, but there was no significant damage to the lower layer. The impact energy was absorbed by the core and the upper layers.
- While the impact resistance of EPS foam was higher in the 10 J impact test, it was determined that the impact resistance and absorption ability of the XPS foam were better in the 20 J and 30 J tests. The mechanical properties of polymer materials can vary with the deformation rate. Therefore, the EPS foam exhibited different behavior at lower and higher deformation rates. However, due to the higher compressive strength and density of the XPS foam compared to the EPS, it yielded better results in high-energy experiments. Additionally, the closed pore structure of the XPS foam enhanced the energy absorption capacity of the sandwich structure.
- The three-point bending test revealed that the bending strength of the sandwich composite with goat wool and the XPS foam was higher.
- XPS foam, with its closed cell structure, increases its elastic deformation capacity and provides better results in energy absorption. The regular structure of the cells ensures that the energy is distributed homogeneously during the transfer of force. In addition, the regular viscoelastic deformation of the XPS foam minimizes energy losses and provides superior performance in both compression and impact tests [65,66].
- EPS foam, with its open and semi-closed cell structure, exhibits a softer structure and low density, which causes its energy absorption capacity to be limited [67]. Due to the weak bond between the cells, more energy is lost during deformation and its resistance to high impact loads decreases. This situation can be clearly observed when the deformation of the core materials (e.g., tearing or collapse) is examined during compression and impact tests.

As a result, XPS foams exhibit more effective performance in applications where high impact resistance and energy absorption capacity are critical, while EPS foams are preferred in applications requiring low loads where lightness and economic use are at the forefront.

In this study, synthetic fibers were compared with synthetic foams. As a suggestion for future researchers, research can be performed using a biodegradable foam matrix reinforced with biodegradable fibers.

## Data Availability

All data generated or analyzed during this study are included in this published article.
